# Emergence of Cutibacterium avidum with *erm*(X) on a Mobile Genetic Element Identical to That of Cutibacterium acnes

**DOI:** 10.1128/mra.00178-23

**Published:** 2023-06-05

**Authors:** Juri Koizumi, Keisuke Nakase, Hidemasa Nakaminami

**Affiliations:** a Department of Clinical Microbiology, School of Pharmacy, Tokyo University of Pharmacy and Life Sciences, Hachioji, Tokyo, Japan; Montana State University

## Abstract

We determined that the Cutibacterium avidum isolate TP-CV302, from a patient with acne vulgaris in Japan, had the macrolide-clindamycin resistance factor *erm*(X) located on Tn*5432*. Although this mobile genetic element (MGE) is well recognized in Cutibacterium acnes, it has not been found in Cutibacterium avidum.

## ANNOUNCEMENT

*Cutibacterium* species inhabit human skin. In addition to Cutibacterium acnes, which exacerbates acne vulgaris, Cutibacterium avidum and Cutibacterium granulosum are present on the skin (1). Antimicrobial treatment for acne has recently led to an increase in antimicrobial-resistant C. acnes strains (2). Besides C. acnes, other *Cutibacterium* species have also acquired antimicrobial resistance (3), with the prevalence of strains that are resistant to macrolides and clindamycin particularly increasing (4, 5). The *erm*(X) gene, which is a macrolide-clindamycin resistance gene, can be horizontally transferred among C. acnes strains via a transposon, Tn*5432* (6). In contrast, C. avidum strains carrying *erm*(X) with IS*Yps3* as the mobile genetic element (MGE) were found in our previous study (3). Therefore, we considered that the *erm*(X) gene could not be transferred between C. acnes and C. avidum. Here, we report the emergence of a C. avidum strain harboring *erm*(X) on Tn*5432*.

C. avidum TP-CV302 was isolated from an acne patient in 2016 (7). The study was approved by the Research Ethics Committee of Tokyo University of Pharmacy and Life Sciences (approval number 16-21). The acne specimen was collected with a sterilized swab and cultured on modified Gifu anaerobic agar medium (Nissui Pharmaceutical, Tokyo, Japan) at 35°C under anaerobic conditions using the AnaeroPack-Anaero (Mitsubishi Gas Chemical Company, Inc., Tokyo, Japan). *Cutibacterium* species were identified using a multiplex PCR that was developed previously (7). The genomic DNA of C. avidum TP-CV302 was extracted using phenol-chloroform-isoamyl alcohol (25:24:1) as reported previously (8). Whole-genome sequencing was performed using an RS II system (Pacific Biosciences, Inc. [PacBio], Menlo Park, CA). Library preparation was performed using the SMRTbell Express template preparation kit v.2.0 (PacBio) according to the manufacturer’s instructions. Single-molecule real-time (SMRT) Link v.11.0.0.146108 was used to align the sequences obtained, with the adapter sequences removed. To filter the reads, Filtlong v.0.2.1 was used to remove reads with <1,000 bases. The data were assembled under default conditions with Flye v.2.9.1-b1780, and the results of the assembled contig graph were checked using Bandage v.0.8.1. The integrity of the assembled genomic data was verified using CheckM v.1.2.2. The whole-genome sequence was obtained through hybrid assembly using Unicycler v.0.4.7. Open reading frame annotation was performed using Prokka v.1.14.5 and DDBJ Fast Annotation and Submission Tool (DFAST) v.1.1.6. NCBI BLAST was used for comparative analysis of nucleotide sequences. The BLAST Ring Image Generator (BRIG) (https://brig.sourceforge.net) was used for comparative analysis (9). Multiple alignments were performed to compare Tn*5432* from C. avidum TP-CV302 and pTP-CU411 (GenBank accession number AP025555.1). Default parameters were used except where otherwise noted.

**FIG 1:**
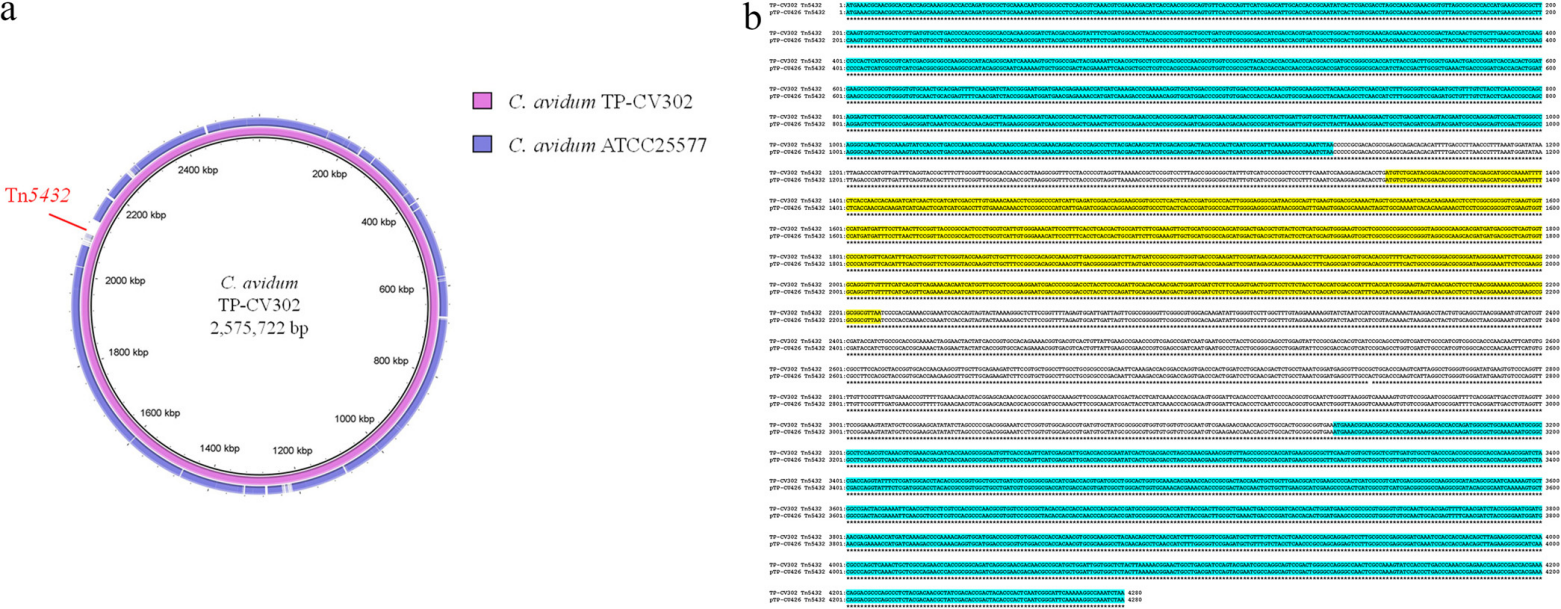
Nucleotide sequence analysis of C. avidum TP-CV302. (a) Nucleotide sequence comparison between C. avidum TP-CV302, which was isolated from a patient with acne, and C. avidum ATCC 25577. The image was generated by using BRIG. (b) Sequences of Tn*5432* from C. avidum TP-CV302 and pTP-CU411 (GenBank accession number AP025555.1) from C. acnes TP-CU411. Both of the sequences contained *erm*(X), and they showed 99.9% identity (4,279/4,280 bp). The blue and yellow shadings indicate IS*1249* and *erm*(X), respectively.

Genome analysis revealed that C. avidum TP-CV302 had a 2,575,722-bp chromosome with a GC content of 63.2% (GenBank accession number AP027369), but no plasmids were found. Comparison with the C. avidum ATCC 25577 chromosome revealed the presence of Tn*5432* with *erm*(X) around 2.1 Mbp (Fig. 1a). C. avidum TP-CV302 showed high-level resistance to macrolides and clindamycin (MICs of ≥256 μg/mL). The Tn*5432* sequence shared 99.9% identity (4,279/4,280 bp) with that of pTP-CU411 (Fig. 1b). Therefore, we discovered that the C. avidum strain had an MGE identical to that of C. acnes.

Our findings indicate that *erm*(X) could potentially be transmitted between C. acnes and C. avidum via Tn*5432*. Therefore, attention should be paid to the prevalence of *Cutibacterium* strains with macrolide and clindamycin resistance acquired through *erm*(X).

### Date availability.

The genome sequence of the C. avidum TP-CV302 chromosome was deposited in NCBI GenBank under the accession number AP027369. The NCBI Sequence Read Archive (SRA) accession number for the raw reads is DRX447838.
